# Regulation of placental development and function by ubiquitination

**DOI:** 10.1186/s10020-025-01268-5

**Published:** 2025-05-23

**Authors:** Xue Wang, Xiaoqing Li, Shanshan Yuan, Zhiju Gu, Zihao An, Qiang Xu, Bin Cao, Yanhua Song, Chao Tang

**Affiliations:** 1 National Clinical Research Center for Child Health, Children’s Hospital Zhejiang University School of Medicine, Hangzhou, China; 2 Institute of Developmental Biology and Molecular Medicine, Fudan University, Shanghai, China; 3 Department of Pathophysiology, Medical School of Nantong University, Nantong, 226001 China; 4 Xinhua Hospital Affiliated to Shanghai JiaoTong University School of Medicine, Shanghai, China; 5 Zhejiang Provincial Center for Disease Control and Prevention, Hangzhou, China

**Keywords:** Placenta, Ubiquitination, Pregnancy diseases, Cellular functions

## Abstract

The proper distribution of nutrients and metabolites between the mother and fetus is one important factor for successful pregnancy. As a bridge, the placenta plays a key role in sensing the nutritional needs of the fetus, coordinating the maternal nutrition supply, and enhancing its nutritional transport capabilities. Imperfect placental development can lead to pregnancy-related disorders such as preeclampsia, recurrent miscarriage, and/or fetal growth restriction, posing risks to both mother and child in the short and long term. However, current understanding of the human placenta remains as a "black box", and its developmental control mechanisms for adaptive pregnant regulation still needs to be elucidated. As one form of post-translational modification (PTM), ubiquitination plays an important role in regulating cellular functions and is regarded as a valuable drug target. Particularly, ubiquitination related to placenta development has been discovered in recent years. Placental development processes closely associated with pregnant complications, such as blastocyst implantation, syncytiotrophoblast cell differentiation, and immune barrier maintenance, have been reported to be affected by ubiquitination. However, the diagnosis and intervention of pregnancy diseases also urgently need to be improved. Thus, aiming to comprehensive summarize and further exploring the molecular mechanism, target and regulatory mechanism of pregnancy complications, we have herein reviewed genes and pathways regulating pregnancy progress and diseases and focusing on ubiquitin-related physiological process in placenta.

**Supplementary Information:**

The online version contains supplementary material available at 10.1186/s10020-025-01268-5.

## Brief introduction and backgrounds

The placenta is a transient organ that serves as a crucial interface between the fetus and the mother, making it indispensable for maintaining the health and well-being of both parties. The formation of the human placenta comprises a series of processes, including embryo implantation, invasion, migration, proliferation, and differentiation of trophoblast cells. The outer wall of the blastocyst is made up of trophoblast cells, which form the earliest and outermost embryonic cell layer. During implantation, the blastocyst invades the endometrial stroma and causes decidualization of the endometrium. At this point, the stromal cells become decidual cells that are larger in size and rich in glycogen and lipids, and surround the blastocyst. The decidua further differentiates into the decidua capsularis that covers the blastocyst, the decidua basalis that lies between the blastocyst and the muscular layer of the uterine wall, and the decidua parietalis (Gellersen and Brosens 2014). The decidua capsularis and decidua parietalis may fuse as the uterine cavity is obliterated. Among them, the orderly arranged ectoderm, mesoderm, and endoderm cells derived from the epiblast constitute the embryonic germ layer, which has a lower degree of differentiation, has mitotic capability, and participates in the fusion of the blastoderm (Gordeev et al. 2024). The outer cells fuse with each other evolved into syncytiotrophoblast, playing a powerful endocrine role. Syncytiotrophoblast is also known spongio-trophoblast, as it forms spongy cavity with villus structure for absorbing the maternal tissue decomposition and transport nutrients to the fetus in its adjacent cavity. The inner has clear boundaries and is composed of a single layer of cubic cells, known as cytotrophoblast, which continuously divides and replenishes into the syncytiotrophoblast. The trophoblast gradually expands outward, eroding the decidua and remodeling the vascular, causing maternal blood to flow through the spongy cavity formed by the fusion of the trophoblast formed by the fusion of the trophoblast (Sharma et al. 2016). While eroding outward, the trophoblast and its derivatives combine with the ectodermal cavity where it covers to form a local papilla that protrudes into the cavity and becomes the primitive villi. Later, the extraembryonic mesoderm grows and stretches into the primary villus, forming the secondary villus structure. Within this structure, the mesoderm differentiates into blood vessels and connective tissues as the middle layer structure, and the projections develop into villi. Finally, the trophoblast layers, which consist of both cytotrophoblast and syncytiotrophoblast, form the villous tree. When the embryo develops the allantois, it outpunches from the hindgut, contributing to the umbilical cord and bringing blood supply (Gauster et al. 2022). This allows the villous trophoblast cells in the allantoic contact area to continue to develop. Subsequently, the placenta progresses into the maternal and fetal compartments, which are delineated by a plentiful vascular network. The rest of the non-contact area's villi stagnate and gradually shrink, forming a smooth villous membrane. The blood vessels of the allantois stalk grow into the umbilical artery and umbilical vein (Arora and Papaioannou 2012). The densely growing villi near the decidua basal area form chorion frondosum, which are clustered into several clusters to form cotyledons with a septum between them, and each cotyledon comprises a network of villous trees. A placenta has 10–20 villous lobes, together these form a series of 30–50 complex three-dimensional villous trees, each centered over the opening of a maternal spiral artery and representing an individual maternal–fetal exchange unit (Jin, et al. 2022). The normal architecture and elaboration of the villous trees plays a pivotal role across gestation, and villous mal-development plays a critical role in pathological pregnancies.

Ubiquitination refers to the process by which ubiquitin, a small molecule (8.5 kDa) composed of 76 amino acids (Chau et al. 1989), acts as an enzyme to select target proteins from intracellular proteins and specifically modify and dispose of them. Ubiquitin is widely present in all eukaryotic cells, and its sequence is highly conserved, with only three amino acids differences from yeast to humans. It is process of a covalent binding of ubiquitin to target and degrading proteins via catalysis of E1 Ubiquitin-activating enzyme, E2 Ubiquitin-conjugating enzymes, and E3 Ubiquitin-ligase enzymes. Briefly, in the presence of ATP, which provides the required energy, the ubiquitin-activating enzyme E1 activates the ubiquitin molecule; Afterwards, the ubiquitin-activating enzyme E1 transfers the activated ubiquitin molecule to the ubiquitin-binding enzyme E2; Finally, ubiquitin ligase E3 attaches the E2-bound ubiquitin to target proteins (Millar et al. 2019), thereby intervening and affecting the fate of the target proteins and regulating intracellular signaling responses. Ubiquitination not only participates in the regulation of protein quantity, but also plays a crucial regulatory role in protein activity, protein–protein interactions, and protein subcellular localization through different lengths of ubiquitin chains (single ubiquitination, multiple ubiquitination, and poly-ubiquitination) and various types of ubiquitin chains (linked through Met1, Lys6, Lys11, Lys27, Lys29, Lys33, Lys48, and Lys63) (Chen et al. 2021a). Due to its diversity and multivalency, ubiquitination is widely involved in various physiological processes, including cell proliferation, apoptosis, autophagy, endocytosis, DNA damage repair, and immune response. And 80–90% of the proteins in the cell are degraded through the ubiquitin pathway (Pickart and Fushman 2004). The classical ubiquitin-controlled protein degradation process involves K48 ubiquitin chain labeling and proteasome (primarily 26S proteasome) specifically recognizing and degrading K48 ubiquitin chain labeling. This degradation system is called the ubiquitin–proteasome system (UPS), which also plays important role in kinds of gestational disorders.

Recent studies have shown that ubiquitination plays a crucial role in placental development, and its disruption may contribute to pregnancy-related disorders. For instance, the classical ubiquitination protein degradation process involves K48 ubiquitin chain labeling and proteasome (primarily 26S proteasome) specifically recognizing and degrading K48 ubiquitin chain labeling. This degradation system, known as the ubiquitin–proteasome system (UPS), plays a significant role in various gestational disorders (Pickart and Fushman 2004). Ubiquitination has important physiological significance. It not only eliminates faulty proteins, but also plays an important regulatory role in the cell growth cycle, DNA replication, and chromosome structure (Benanti 2012; Martin-Rufo et al. 2022; Feng et al. 2022). Therefore, they are involved in the development and progression of many diseases and are potential therapeutic targets in modern medicine. To better understand the regulation of the functional activity of the placenta, a"black box"that influences pregnancy outcomes, we have reviewed ubiquitination-related physiological processes in placenta and summarized related genes and pathways. This review aims to provide a deeper understanding of ubiquitination and its implications for maternal–fetal health. By synthesizing emerging evidence and leveraging new technologies, we explore how ubiquitination influences placental function, offering insights that underscore the importance of this PTM in the biology of pregnancy.

## Ubiquitination regulates the physiological function of placenta

### Nutrients and substances exchange related ubiquitination

The placenta is the primary interface and the essential organ responsible for nutrient and substance exchange between the mother and fetus. The oxygen, free fatty acids, glucose, amino acids, vitamins, and electrolytes needed for fetal growth and development can be transported through the placenta into the fetal blood by simple diffusion and active transport (Jin et al. 2021). The placenta provides metabolic support rather than directly breaking down complex structures enzymatically for fetal nutrition. On one hand, it produces various enzymes that can decompose complex structures into simple substances, or synthesize glycogen, protein, and cholesterol from simple structures, providing nutrients for the fetus. On the other hand, fetal metabolites, such as carbon dioxide, uric acid, and urea, are also transported into the maternal blood through the placenta and are consequently eliminated from the body along with the mother’s.

The ubiquitination of proteins plays a key role in the aforementioned physiological processes (Summarized in Table 1). For example, murine trophoblasts-derived exosome-enriched extracellular vesicles (ExoE-EVs) are associated with pregnancy and orchestrate physiological processes in the placenta. The ExoE-EVs facilitate intercellular communication by carrie X-chromosome miRNAs, which target ubiquitin-mediated proteolysis and are predicted to affect maternal–fetal immune interactions and the differentiation of syncytial-like cells (Stefanski et al. 2019). Sodium-coupled neutral amino acid transporter 2 (SNAT-2), a target of rapamycin (mTOR) signaling, regulates system amino acid transport. In placentas with fetal growth restriction (FGR), the ubiquitin ligase, Neural precursor cell-Expressed Developmentally Down-regulated protein 4–2 (NEDD4-2), up-regulates the ubiquitination of SNAT2, resulting in the suppressed activity of mTORC1/2 (Chen et al. 2015). On the other hand, epigenomic modifications, which are chemical alterations on chromosomal DNA and proteins, can regulate the exchange of metabolites in the placenta. This, in turn, can influence ubiquitination of associated proteins, ultimately impacting fetal development. For instance, SIAH3, a member of the E3 ubiquitin protein ligase family, experiences hypomethylation in placentas with elevated cadmium (Cd) levels, consequently impacting protein ubiquitination in the placenta and subsequently influencing maternal health and fetal development (Mohanty et al. 2015). PKA treatment increases GCMa acetylation and protects it from ubiquitination with a concomitant increase in transcriptional activity (Chang et al. 2005). During chronic hypoglycemia, the fetus of sheep exhibits increased rates of lysine flux and oxidative metabolism, accompanied by elevated muscle-specific ubiquitin ligases FBXO32 and RFP28, as well as greater concentrations of 4E-BP1 (Limesand et al. 2009), which supports the finding that hypoglycemia does not cause changes in fetal protein accumulation or synthesis.

**Table 1:** Ubiquitination regulation related to placental function of transport, immunity and endocrine homeostasis

Gene, PTMs, or gene family	Regulate target	Pathological features of placenta	Species/Function
SNAT-2	mTOR signaling regulated ubiquitination	Placental amino acid transport is decreased; intrauterine growth restriction	Human/Transport
X-chromosome miRNAs	Predicted to target ubiquitin-mediated proteolysis and intracellular signaling pathways	Carried by exsome, plays vitrol role ExoE-EVs, particularly from the X chromosome cluster	Mice/Transport
SIAH3	Hypomethylated in high Cd placentas	Infant sex-specific associations of placental Cd with genome-wide DNA methylation in the placenta	Human/Transport
GCMa	PKA protect GCMa from ubiquitination and increases the TAD stability	Regulates trophoblastic fusion	Human/Transport
FAU	A gene encoding MNSF-β, ubiquitously expressed ubiquitin-like protein interacting with RC3H1	Regulates TNFα production in decidual macrophages; RSA	Human/Immunity balance
MAGE-A3	Acts as a cellular master regulator by stimulating E3 ubiquitin ligase TRIM28	Regulation of various cellular targets and an attractive target for vaccine-based immune therapy	Human/Immunity balance
Pellino (Peli)1	E3 Ubiquitin Protein Ligase 1, which mediated cytokine and chemokine responses and	Induced cell death in placental trophoblasts and human neural stem cells	Mice/Immunity balance
UHRF1	Mediate histone ubiquitination, and activating the MyD88/NF-κB signaling pathway	Increasing the CXCL2/IL-1β in the trophoblasts, promoting the polarization of decidual macrophage differentiation to M1 phenotype; RSA	Human, Mice/Immunity balance
ISG15	Functional ubiquitin homologue gene	Regulated by mononucleate cells and IFNT and affect endometrial stroma; Implantation failure	Sheep/Immunity balance
SOX4	SOX4-PGR-HERC4 axis mediated degradation	Regulated WNT5 A, WNT4, IL-11, BMP2, and so on; human endometrial decidualization; Implatation failure	Human/Endocrine homeostasis
phospho-CREB (p-CREB)	Interacts with p-CREB to prevent its ubiquitination via FOXO1	Aldosterone biosynthesized in endometrial gland during mid-secretory phase promotes decidualization	Human/Endocrine homeostasis
RGS2	Suppressing USP14 mediated deubiquitination of HAND1	Estradiol was increased by regulated HAND1; Pre-eclampsia	Human/Endocrine homeostasis
BAP1	BAP1/ASXL complex; form the polycomb repressive deubiquitinase (PR-DUB) complex	EMT progression; early placentation	Human/Endocrine homeostasis

### Immune defense function related ubiquitination

The placental barrier separates fetal blood from maternal blood to avoid or reduce the impact of harmful factors on the fetus. The defensive function of the placenta can prevent macromolecules in the maternal blood from entering the fetal blood circulation. On one hand, since the second trimester, immunoglobulin G (IgG) antibodies contained in the maternal blood increased, through term, most antibodies being acquired during the third trimester through the placenta (Simister 2003). On the other hand, it prevents large molecules such as bacteria and pathogens in the maternal blood from penetrating the placenta directly into the fetal blood circulation (Brett et al. 2014). However, certain pathogens may cross the placenta in cases of placental barrier damage or infection (Chenge, et al. 2023). Additionally, the placenta's ability to resist barbiturates (Browne et al. 2014), morphine (Lyu et al. 2024), and various foreign viruses such as toxoplasma gondii (Hubal et al. 2025) and mycoplasma (Pan et al. 2025) is delicate.

Many proteins are ubiquitinated to participate in immune response, triggering and/or assisting in the normal function of placenta protection (Goor et al. 2020) (See Table 2). For example, the expression of monoclonal nonspecific suppressor factor-β (MNSF-β), a ubiquitously expressed ubiquitin-like protein, is significantly increased in decidual macrophages of recurrent spontaneous abortion (RSA) patient. RSA is a pregnancy complication usually defined as three or more spontaneous abortions prior to 20–28 weeks gestation. The etiology of RSA is complex and diverse. In addition to genetic factors, autoimmune abnormalities, thrombophilia, and endocrine factors may be the causes of RSA. The aberrantly increased MNSFβ expression in decidual macrophages promotes TNFα production via its interaction with RC3H1 (Zhen et al. 2021), ultimately leading to immune dysfunction in the maternal–fetal interface and resulting in pregnancy loss. Melanoma Associated Antigen A3 (MAGE-A3), a protein exclusively expressed in testes and placenta, has been found to stimulate E3 ubiquitin ligase Tripartite Motif-containing protein 28 (TRIM28). It regulates the cellular energy sensor AMP-activated protein kinase and has consequently been regarded as a promising target for vaccine-based immune therapy (Schafer et al. 2021). Ubiquitination may also play a role in the disruption of placental function caused by infection or immune dysregulation. For instance, Pellino (Peli), an E3 ubiquitin ligase found in placental trophoblasts, mediates inflammatory cytokine and chemokine responses, as well as induce cell death, in placental trophoblasts and human Neural Stem Cells. Pregnant mice infected with the Zika virus and lacking Peli1 signaling display reduced placental inflammation and tissue damage, ultimately leading to a milder manifestation of congenital abnormalities (Luo et al. 2020). Ubiquitin like with PHD and ring finger domains 1 (UHRF1) is proved to be as a key regulator on the trophoblasts and their cross-talk with local immune cells, has been demonstrated as a potential approach for RSA intervention (Liu et al. 2022a). Similarly, Interferon-stimulated gene 15 (ISG15) is regulated by mononucleate cells and IFNT, affecting endometrial stroma and contributing to implantation failure (Joyce et al. 2005).

**Table 2:** Pathological features and ubiquitination related gene involved in embryo implantation

Gene Name	Mechanism	Pathological features of placenta	Species
Sox4	Repressing E3 ubiquitin ligase HERC4	Dysregulated decidualization of stromal cells; Implantation failure	Sheep
SKP2	A key component of the SCF-type E3 ubiquitin ligase complex	Reduced in the decidual tissues and down-regulated GLUT1; RSA	Human
USP7	Deubiquitinating directly interacted with the EZH2 and regulated the Wnt/β-catenin signaling pathway	Regulate trophoblast proliferation, apoptosis, migration, and invasion; Downregulated in the placental villous tissues, RSA	Human
Smurf2	An E3 ubiquitin ligase		Rhesus monkey endometrium
SPOP	An adapter of E3 ligases of ubiquitination	Regulates endometrial stromal cell decidualization and affected by hormones	Mice
MAP3 K4	Promotes HDAC6 ubiquitination and degradation	Maintaining the epithelial state, deacetylating histones on epithelial gene promoters, promoting the dissolution of tight junctions; epithelial-to-mesenchymal transition	Mice
ISG15	Functional ubiquitin homologue gene	Regulated by mononucleate cells and IFNT and affect endometrial stroma; Implantation failure	Sheep
FAU	A gene encoding MNSF-β, a ubiquitously expressed ubiquitin-like protein promote TNFα production via its interaction with RC3H1	Significantly increased in decidual macrophages (dMϕ); RSA patient	Human
UHRF1	Mediate histone ubiquitination, and activating the MyD88/NF-κB signaling pathway	Increasing the pro-inflammatory TH-1 type chemokine/cytokines CXCL2/IL-1β in the trophoblasts, promoting the polarization to M1 phenotype; RSA	Human, Mice

In conclusion, the contribution of ubiquitination to the placental immune defense primarily relies on the anatomical characteristics of the decidua and the intercellular communication mechanism of immune cells. To further understand the role of ubiquitination, investigating its involvement in the following physiological processes of placental immune development is necessary. A successful pregnancy enables the embryo and fetus to develop within the uterus while keeping the mother's immune system. Uterine natural killer cells, immature dendritic cells, T cells, and macrophages contribute to creating a favorable uterine environment for a successful pregnancy (Andreescu, et al. 2025). By studying the role of ubiquitin in the communication and activation of immune cells, we can uncover related pathological mechanisms, thereby laying a scientific foundation for clinical diagnosis and treatment.

### Endocrine homeostasis-related ubiquitination

The placenta is an endocrine organ that secretes various hormones, cytokines, and neurotransmitters, which play an important role in maintaining normal pregnancy and regulating fetal development. In the early stages of pregnancy, human chorionic gonadotropin and prolactin secreted between the second and eighth weeks, promoting fetal growth and development while also participating in enhancing the secondary growth and development of the mother's mammary glands. In addition, the synthesis and secretion of cytokines such as epidermal growth factor, nerve growth factor, and interleukins by the placenta not only contribute to the healthy growth of the fetus, but also can play an irreplaceable role in early trophoblast cells'invasion, proliferation and differentiation of cytotrophoblast, as well as in maternal tolerance and non-rejection of the fetus through ubiquitination. As an instance, SOX4, acting as a pivotal regulator, regulates the stability of progesterone receptor (PGR) by suppressing the degradation mechanism of E3 ubiquitin ligase HERC4. Furthermore, several genes associated with secretion, fetal development, and stem cell differentiation (such as WNT5 A, WNT4, IL-11, and BMP2) have been found to be down-regulated significantly following the silencing of SOX4 (Huang, et al. 2022). In another case, in order to maintain early pregnancy and provide crucial nutrients and an immune environment for placental formation, the endometrium must undergo decidualization. Aldosterone biosynthesized in endometrial gland during mid-secretory phase promotes decidualization via activating MR/LKB1/p-AMPK/PDK4/p-CREB/FOXO1 signaling pathway, in which PDK4 interacts with p-CREB to prevent its ubiquitination and facilitate decidualization via FOXO1 (Li et al. 2020). Ubiquitination related endocrine gene also associated to pregnancy diseases. For example, RGS2 inhibits the deubiquitination of HAND1 by ubiquitin-specific protease 14 (USP14), leading to the restoration of HAND1-induced trans-inactivation of the *aromatase* gene and subsequent elevations in estradiol levels, which may contribute to pre-eclampsia (Tang et al. 2023). Several pregnancy complications such as miscarriage, pre-eclampsia, placenta accreta, and FGR are underpinned by a primary defect in epithelial-mesenchymal transition (EMT) progression. BAP1, a catalytic subunit of a deubiquitinase complex, suppressed EMT progression dependent on the binding to additional sex comb-like (ASXL1/2) proteins to form the polycomb repressive deubiquitinase (PR-DUB) complex in mouse trophoblasts. While its dependency on additional sex comb-like (ASXL1/2) proteins binding is not directly relevant, BAP1 has been associated with pregnancy complications and its protein levels significantly down-regulated during EMT (Perez-Garcia, et al. 2021).

In conclusion, ubiquitination plays a crucial role in the network of fetal, maternal, and placental. However, despite the importance of this process, relevant research is still limited. In addition to gene functions, we were surprised to discover that metabolites, PTMs, and certain non-coding RNAs can regulate the processing of the placenta through ubiquitination. As metabolomics and epigenomics continue to advance, we anticipate that more physiological processes related to ubiquitination will be identified. Meanwhile, it should be noted that due to the differences between the human placenta and those of other organisms, the ubiquitination mechanisms may also vary. From this perspective, as research on pregnancy-related ubiquitination deepens, it can also reveal scientific theories related to species evolution, which is also very meaningful.

## Ubiquitination regulated pregnancy maintenance and diseases

**Fig. 1:**
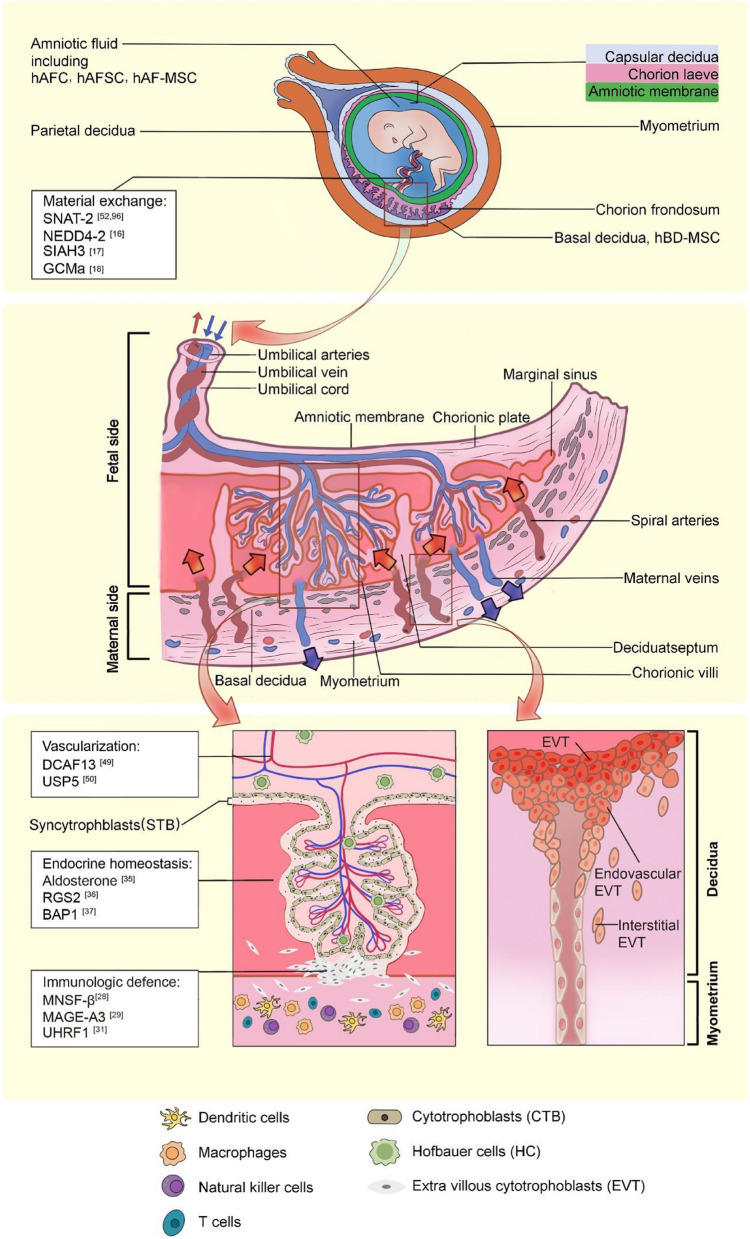
A schema chart showing the relationship between the ubiquitin-related genes and human placenta

Early defects and dysfunction in placental development are the primary causes of various common pregnancy-related diseases, such as recurrent miscarriage, fetal intrauterine growth restriction, preeclampsia, and preterm birth (Vishnyakova et al. 2019; Vishnyakova et al. 2021). Mammalian placental cells are vulnerable to mitochondrial dysfunction, oxidative damage, and the buildup of cross-linked ubiquitinated proteins, which are frequent causes of placental dysfunction. The ubiquitin–proteasome degradation pathway plays a crucial regulatory role in preserving the dynamic equilibrium of proteins and cellular function within the body. We herein have summarized the ubiquitin-related genes and mechanisms involved in the progress of pregnancy and various diseases (Fig. 1).

### Implantation failure

Placental invasion is the initial step after embryo implantation, including sequential pre-contact, apposition, adhesion, invasion and penetration of the decidua, followed by the formation of chorionic villi (Ochoa-Bernal and Fazleabas 2020). Implantation causes the secretory-phase endometrium to become thicker and more abundant for blood supply. The stromal fibroblasts further expand and accumulate glycogen, preparing to differentiate into decidual cells with specialized secretory functions (Gellersen and Brosens 2014). During this process, differentiation of endometrial stromal cells for pregnancy present morphological and physiological changes to adapt to pregnancy. Ubiquitination plays a critical role in regulating cell differentiation and signaling pathways. Dysregulated decidualization is always associated with failure of pregnancy. Recent evidence has shown that dysregulated SOX4-HERC4-PGR axis is a potential cause of defective decidualization and recurrent implantation failure in in vitro fertilization patients (Huang, et al. 2022). The F-box protein S-phase kinase associated protein 2 (SKP2) is a key component of the SCF-type E3 ubiquitin ligase complex, which is significantly reduced in the decidual tissues of RSA patients and suppresses the expression of its downstream target, GLUT1 (Lv et al. 2021). The expression of SPOP, an adapter of E3 ligases of ubiquitination, in mouse uteri during early pregnancy could be controlled by hormones and regulates endometrial stromal cell decidualization, indicating the role of ubiquitination in embryonic implantation.

Trophoblasts are placental stem cells and play crucial roles in maternal–fetal crosstalk. The first developmental EMT occurs in trophoblast cells during implantation, where epithelial trophoblast cells in the trophectoderm transition into invasive giant cells (Thiery et al. 2009). Adequate trophoblast migration and invasion are essential for successful placental development and implantation (Vento-Tormo et al. 2018). The expression of ubiquitin-specific protease 7 (USP7), a member of the deubiquitinating enzyme family, is downregulated in the placental villous tissues of RSA patients. Recent studies have revealed that USP7-related deubiquitination directly interacts with the enhancer of zeste homolog 2 (EZH2) and regulates trophoblast proliferation, apoptosis, migration, and invasion through the Wnt/β-catenin signaling pathway in trophoblasts in the maternal–fetal interface (Zhou et al. 2023; Yang et al. 2007). Smad ubiquitin regulatory factor 2 (Smurf2) is an E3 ubiquitin ligase that triggers the ubiquitin-dependent degradation of the SMAD7-mediated transforming growth factor beta (TGF-beta) receptor (Kavsak et al. 2000). In hesus monkey endometrium and placenta, Smurf2 may play specific roles in glandular secretion, trophoblastic cell invasion, and placentation through TGF-beta signaling pathway during early pregnancy (Yang et al. 2007). Importantly, the first epithelial-to-mesenchymal transition (EMT) occurs in trophoblast stem cells during implantation. Mobley et al*.* find that HDAC6 is regulated by MAP3 K4 during trophoblast stem cell differentiation and EMT. They prove that MAP3 K4 promotes HDAC6 ubiquitination and degradation, maintaining the epithelial state, and HDAC6 directly deacetylates histones on epithelial gene promoters such as *claudin6* and *occludin*, promoting the dissolution of tight junctions (Mobley et al. 2017). Ubiquitination of immune cells also plays critical roles in implantation. For example, interferon stimulated gene 15 (ISG15) is a ubiquitin-like protein whose expression and conjugation to targets (ISGylation) is induced by multiple factors such as infection, DNA damage, and ischemia. ISG15 conjugation/ISGylation in decidua is involved in one or more of physiological function of decidual. The mononuclear cells of the trophectoderm secrete IFNT, which can stimulate the expression of functional ubiquitin homologue gene *ISG15* in the endometrial stroma of sheep (Joyce et al. 2005). The disruption of aforementioned MNSF-β and UHRF1 related to the immune balance also contribute to implantation failure and pregnancy loss (Zhen et al. 2021).

### Vascularization of the placenta and eclampsia or preeclampsia

Vascularization of the placenta is a critical developmental process that ensures fetal viability. Preeclampsia (PE) is a complication of pregnancy that occurs after 20 weeks of gestation and is highly related to placental vascularization. PE patients exhibit symptoms of high blood pressure, high levels of protein in urine that indicate kidney damage (proteinuria), or other signs of organ damage. In severe cases, PE can progress to eclampsia, accompanied by convulsions, posing a threat to the lives of both the mother and the fetus. Most ubiquitylation studies on the pathogenesis of PE or eclamptic placenta and ubiquitination have been reported (Summarized in Table 3). The ubiquitin ligase Ankyrin repeat SOCS box-containing 4 (ASB4) promotes embryonic stem cell differentiation to vascular lineages and is highly expressed early in placental development. The transcriptional regulator inhibitor of DNA binding 2 (ID2) negatively regulates vascular differentiation during development and is a target of many ubiquitin ligases. ASB4 mediates vascular differentiation in the placenta by degrading ID2, and deletion of *Asb4* in mice induces a pathology that phenocopies human pre-eclampsia, including hypertension and proteinuria (Townley-Tilson et al. 2014). The Cullin 4-RING E3 ubiquitin ligase (CRL4) complex ubiquitinates and degrades substrates, while DDB1 and CUL4-associated factor 13 (DCAF13) is a component and substrate receptor of this complex, which recognizes and recruits the complex different substrates. The DCAF13 levels in the decidua of PE patients are significantly lower than that of normal pregnant women, and *Dcaf13* knockout mice fails to undergo decidualization (Yan et al. 2022). USP5 is lowly expressed in the placentas of PE patients as well as in hypoxia/reoxygenation-induced trophoblast cells. USP5 promotes the proliferation of trophoblast cells via activating Wnt/β-catenin signaling pathway (Zhang et al. 2021), and the downstream signals, such as c-Myc and Cyclin D1. A more recent report shows that RGS2 contributes to PE via USP14/HAND1-regulated estradiol biosynthesis (Tang et al. 2023).

**Table 3:** Ubiquitination related gene involved in pregnant diseases

Gene, gene family, or protein complex	Mechanism	Pathological features of placenta	Species/Disorder
ASB4	Activate ubiquitin ligases and degradation of ID2 (negative vascular differentiation)	Promoted embryonic stem cell differentiation to vascular lineages and highly expressed during early placental development	Mice/PE
CRL4	E3 ubiquitin ligase	DDB1 and CUL4-associated factor 13 (DCAF13) complex levels in the decidua of PE patients is significantly lower; knocking-out of DCAF13 causese decidualization falure	Human;Mice/PE
USP5	A deubiquitinase	Lowly expressed in the placentas of PE patients as well as in hypoxia/reoxygenation-induced trophoblast cells	Human/PEa
RGS2	Suppressing USP14 mediated deubiquitination of HAND1	Estradiol was increased by regulated HAND1; Pre-eclampsia	Human/PE
miR-135a-5p	Target E3 ubiquitin protein ligase β-TrCP	Regulates EMT of trophoblast cells	Human/PE
X-chromosome miRNAs	Predicted to target ubiquitin-mediated proteolysis and intracellular signaling pathways	Carried by exsome, plays vitrol role ExoE-EVs, particularly from the X chromosome cluster	Mice/PE
SNAT-2	mTOR signaling regulated ubiquitination	Placental amino acid transport is decreased; intrauterine growth restriction	Human/PE
A20	A ubiquitin modifying enzyme	IUGR can reduce the expression of A20 protein in lung tissue of newborn rats	Mice/IUGR
CUL7	Participating ubiquitin–proteasome system	Up-regulated up to 10 times in IUGR and 15 times in preeclampsia associated with IUGR	Human/IUGR&PE
UBE variants	The ubiquitin-conjugating enzymes	Differentially expressed and associated with GDM	Human/GDM
UBE2E2	The ubiquitin-conjugating enzymes	UBE2E2 single-nucleotide polymorphisms rs7612463 showed a significant association with GDM	Human/GDM
GβL	A ubiquitination-dependent mammalian target of rapamycin	Maternal hyperglycemia inhibits pulmonary vasculogenesis during fetal lung development by promoting GβL-ubiquitination-dependent mTORC1 assembly	Mice/GDM

MicroRNAs can also regulate the ubiquitination in placenta and thus contributes to PE. For example, miR-135a-5p is targeted and regulated by β-transducin repeat containing E3 ubiquitin protein ligase (β-TrCP) in the placental of PE patients (Wu et al. 2020). In molecular biological experiments, down-regulated E-cadherin levels and increased N-cadherin, Vimentin, and β-catenin levels that are induced by miR-135a-5p overexpression are attenuated by β-TrCP overexpression. In addition, X-chromosome miRNAs carried by ExoE-EVs are predicted to target ubiquitin-mediated proteolysis and intracellular signaling pathways (Stefanski et al. 2019), however, their functions and the correlation with PE remain to be studied.

### Other pregnant diseases

Some other common pregnancy complications have also been reported to be related to ubiquitination. Intrauterine growth restriction (IUGR), also known as FGR as previously mentioned, is when the fetal weight is estimated to be below the 10 th percentile for its gestational age. The pathogenesis of IUGR is complex, with most patients experiencing placental hypoperfusion and hypoplasia, which are closely linked to ubiquitination. For example, the activity of mTORC1 and mTORC2 is decreased whereas the protein expression of the ubiquitin ligase NEDD4-2 (+ 72%, *P* < 0.0001) and the ubiquitination of SNAT-2 (+ 180%, *P* < 0.05) are increased in homogenates of FGR placentas (Gascoin-Lachambre et al. 2010). Cullins constitute a family of seven proteins involved in cell scaffold and in selective proteolysis via the ubiquitin–proteasome system, the *CUL7* gene is up-regulated up to 10 times in FGR and 15 times in preeclampsia associated with FGR (Bari et al. 2016). Gestational diabetes mellitus (GDM) is a condition in which a hormone made by the placenta prevents the body from using insulin effectively. Glucose builds up in the blood instead of being absorbed by the cells. A transcriptomic profiling analysis of trophoblast isolated from gestational diabetes mellitus (GDM) defined 8 ubiquitin-conjugating enzymes (UBE) splice variants (UBE2D3 variants 1, 3, 4, 5, 6, 7, 9 and UBE2V1 variant 4) are associated with increased maternal fasting plasma glucose (Kim et al. 2013). In another research, the genetic variants of ubiquitin-conjugating enzyme UBE2E2 are also reported to be associated with GDM (Xu et al. 2023). Ubiquitination may also trigger other complications from pregnancy disorders. For example, the decreased ubiquitin modifying enzyme A20 is associated with hyper-responsiveness to ovalbumin challenge following FGR (Luo et al. 2023). The maternal hyperglycemia inhibits pulmonary vasculogenesis during mouse fetal lung development by promoting G protein beta subunit-like protein (GβL) ubiquitination-dependent mammalian target of rapamycin assembly (Gascoin-Lachambre et al. 2010). With the advancement of omics technology, an increasing number of ubiquitination mechanisms associated with pregnancy complications will be discovered, holding great promise for the development of therapeutic approaches for these diseases.

Our review summarizes many ubiquitination processes related to pregnancy complications, potentially providing new insights for drug development. The placenta is a complex tissue composed of various cells and structures, making it a crucial component. Therefore, studying the ubiquitination processes on the development of a specific group of cells during a particular period by utilizing spatial omics methods may provide more insight in to explanation of diseases. In addition, The ubiquitin mechanism can be harnessed for targeted protein degradation techniques, such as molecular glue (Schreiber 2021) and proteolysis-targeting chimeras (Yang et al. 2019), which are key focuses in the pharmaceutical industry.

## Identification of ubiquitination-associated protein regulation in gestational disorders by Mendelian randomization analysis

To additionally figure out the ubiquitination-associated protein regulation in gestational disorders, we carried out the Mendelian randomization analysis by using database when ubiquitination related plasma proteins as outcome and pregnant diseases as exposure. 85 ubiquitination related plasma proteins were screened (Table 4), which were used as expose factors in subsequent Mendelian randomization analysis. Four major pregnancy related diseases from a Finnish database were set as outcomes to explore the potential ubiquitination related molecular mechanisms of action in perinatal diseases. The specific information and download links for the outcomes can be found in Table 5. The positive results obtained from the screening were presented in Table 6, and the corresponding single nucleotide polymorphisms (SNPs) used for the exposure factors in the positive results are summarized in Table S1.

**Table 4:** Information regarding the ubiquitination related plasma proteins utilized as exposures in Mendelian randomization analysis

Expose data (Seq id)	Protein name	Paticipated pathway related to ubiquitination				
		**Deubiquitination**	**Ub-specific processing proteases**	**Protein ubiquitination**	**Ubiquitination Cascade**	**Regulation of FZD by ubiquitination**
16825_20	ATXN3	⚫				
12925_105	AXIN2	⚫	⚫			
13977_28	BARD1	⚫				
13032_1	BECN1	⚫	⚫			
10046_55	BIRC2	⚫	⚫		⚫	
15319_226	CCNA1	⚫	⚫			
15574_37	CCNA2	⚫	⚫			
13089_6	HIF1 A	⚫	⚫			
3197_70	IDE	⚫	⚫			
12551_3	ITCH				⚫	
16304_6	LGR4					⚫
16296_43	LGR5					⚫
18392_19	MAT2B	⚫	⚫			
13228_75	MDM2	⚫	⚫		⚫	
13229_20	MDM4	⚫	⚫			
9315_16	MUL1	⚫	⚫			
11536_9	MYSM1	⚫				
12493_42	OTUB2	⚫			⚫	
12480_9	OTUD5	⚫				
19364_163	PCNA			⚫		
8300_82	PEX14			⚫		
7880_9	PSMA1	⚫	⚫			
4280_47	PSMA2	⚫	⚫			
18925_24	PSMA5	⚫	⚫			
12460_18	PSMA7	⚫	⚫			
12612_37	PSMB1	⚫	⚫			
18339_207	PSMB3	⚫	⚫			
18340_2	PSMB4	⚫	⚫			
12580_7	PSMB5	⚫	⚫			
10530_8	PSMB6	⚫	⚫			
18942_11	PSMB9	⚫	⚫			
18385_4	PSMD10	⚫	⚫			
13572_43	PSMD11	⚫	⚫			
13568_30	PSMD4	⚫	⚫			
10716_35	PSMD5	⚫	⚫			
3898_5	PSMD7	⚫	⚫			
5918_5	PSME1	⚫	⚫			
17694_32	PSME2	⚫	⚫			
16887_29	PTRH2	⚫	⚫			
12522_6	RAD23B	⚫				
8970_9	RIPK2	⚫				
6510_56	RNF128	⚫	⚫			
11401_181	RNF146	⚫	⚫			
14120_2	RNF43					⚫
16614_27	RSPO1					⚫
8409_3	RSPO2					⚫
13094_75	RSPO3					⚫
8464_31	RSPO4					⚫
9838_4	SMAD1	⚫	⚫			
10364_6	SMAD2	⚫	⚫			
10363_13	SMAD3	⚫	⚫			
12022_12	SMAD4	⚫	⚫			
13985_12	SMURF2	⚫	⚫			
19187_21	STAMBP	⚫	⚫			
12401_3	STAMBPL1	⚫				
11350_30	STUB1				⚫	
10703_203	SUDS3	⚫	⚫			
18270_10	TAF10	⚫	⚫			
2333_72	TGFB1	⚫				
5133_17	TGFBR2	⚫				
14009_65	TNFAIP3	⚫				
9907_216	TNKS	⚫	⚫			
6123_69	TP53	⚫	⚫			
6651_74	UBB	⚫	⚫	⚫	⚫	⚫
17743_14	UBE2A			⚫	⚫	
9865_40	UBE2B			⚫	⚫	
12556_7	UBE2C			⚫	⚫	
19247_1	UBE2D1	⚫		⚫	⚫	
18842_24	UBE2D2			⚫	⚫	
19280_29	UBE2D3			⚫	⚫	
14326_4	UBE2E1			⚫	⚫	
18213_30	UBE2F				⚫	
9199_6	UBE2G2			⚫	⚫	
3874_8	UBE2L3			⚫	⚫	
19190_4	UBE2L6				⚫	
19111_10	UBE2M				⚫	
12400_25	UBE2T			⚫	⚫	
11226_16	UBE3 A				⚫	
5019_16	UCHL1	⚫				
19161_1	USP15	⚫	⚫			
12681_63	USP21	⚫	⚫			
9215_117	USP25	⚫	⚫			
13450_49	USP8	⚫	⚫			⚫
13236_25	WNT3 A					⚫
14122_132	ZNRF3					⚫

**Table 5:** Information regarding the pregnant diseases utilized as outcomes in Mendelian randomization analysis

GWAS id	Pregnant disease	Number of cases	Number of controls	Download linkage
finn-b-O15_PRE_OR_ECLAMPSIA	Pre-eclampsia (PE)	8818	234034	https://storage.googleapis.com/finngen-public-data-r11/summary_stats/finngen_R11_O15_PRE_OR_ECLAMPSIA.gz
finn-b-GEST_DIABETES	Gestational diabetes mellitus (GDM)	16802	237816	https://storage.googleapis.com/finngen-public-data-r11/summary_stats/finngen_R11_GEST_DIABETES.gz
finn-b-O15_POOR_FETGRO	Fetal growth restriction (FGR)	4617	250001	https://storage.googleapis.com/finngen-public-data-r11/summary_stats/finngen_R11_O15_POOR_FETGRO.gz
finn-b-O15_PRETERM	Preterm birth (PTB)	10350	194563	https://storage.googleapis.com/finngen-public-data-r11/summary_stats/finngen_R11_O15_PRETERM.gz

**Table 6:** Mendelian randomization estimates of the effect of ubiquitination related plasma proteins on pregnant diseases

Expose/Plasma protein	Indexs	Analyze methods	P or Q valuse	Outcome/Pregnant disease
ATXN3	Mendelian Randomization result	MR Egger	0.271	GDM
		Weighted median	0.042	
		Inverse variance weighted	0.027	
		Simple mode	0.383	
		Weighted mode	0.122	
	Heterogeneity tests	MR Egger	0.649	
		Inverse variance weighted	0.798	
	Horizontal Pleiotropy	MR-Egger regression intercept	0.907	
		MR-PRESSO global test	0.904	
BARD1	Mendelian Randomization result	MR Egger	0.950	GDM
		Weighted median	0.258	
		Inverse variance weighted	0.035	
		Simple mode	0.148	
		Weighted mode	0.583	
	Heterogeneity tests	MR Egger	0.072	
		Inverse variance weighted	0.136	
	Horizontal Pleiotropy	MR Egger regression intercept	0.773	
		MR-PRESSO global test	0.296	
CCNA2	Mendelian Randomization result	MR Egger	0.680	FGR
		Weighted median	0.071	
		Inverse variance weighted	0.042	
		Simple mode	0.307	
		Weighted mode	0.256	
	Heterogeneity tests	MR Egger	0.755	
		Inverse variance weighted	0.946	
	Horizontal Pleiotropy	MR Egger regression intercept	0.927	
		MR-PRESSO global test	-	
MDM4	Mendelian Randomization result	MR Egger	0.799	PTB
		Weighted median	0.001	
		Inverse variance weighted	0.001	
		Simple mode	0.093	
		Weighted mode	0.083	
	Heterogeneity tests	MR Egger	0.846	
		Inverse variance weighted	0.980	
	Horizontal Pleiotropy	MR Egger regression intercept	0.973	
		MR-PRESSO global test	-	
PSMA7	Mendelian Randomization result	MR Egger	0.206	FGR
		Weighted median	0.027	
		Inverse variance weighted	0.035	
		Simple mode	0.173	
		Weighted mode	0.109	
	Heterogeneity tests	MR Egger	0.673	
		Inverse variance weighted	0.718	
	Horizontal Pleiotropy	MR Egger regression intercept	0.503	
		MR-PRESSO global test	0.781	
PSMB6	Mendelian Randomization result	MR Egger	0.818	PE
		Weighted median	0.106	
		Inverse variance weighted	0.049	
		Simple mode	0.339	
		Weighted mode	0.255	
	Heterogeneity tests	MR Egger	0.736	
		Inverse variance weighted	0.855	
	Horizontal Pleiotropy	MR Egger regression intercept	0.732	
		MR-PRESSO global test	-	
RSPO1	Mendelian Randomization result	MR Egger	0.097	GDM
		Weighted median	0.012	
		Inverse variance weighted	0.047	
		Simple mode	0.097	
		Weighted mode	0.049	
	Heterogeneity tests	MR Egger	0.304	
		Inverse variance weighted	0.311	
	Horizontal Pleiotropy	MR-Egger regression intercept	0.386	
		MR-PRESSO global test	0.434	
RSPO3	Mendelian Randomization result	MR Egger	0.515	FGR
		Weighted median	0.078	
		Inverse variance weighted	0.048	
		Simple mode	0.220	
		Weighted mode	0.168	
	Heterogeneity tests	MR Egger	0.702	
		Inverse variance weighted	0.810	
	Horizontal Pleiotropy	MR Egger regression intercept	0.698	
		MR-PRESSO global test	0.829	
STAMBPL1	Mendelian Randomization result	MR Egger	0.815	GDM
		Weighted median	0.056	
		Inverse variance weighted	0.012	
		Simple mode	0.191	
		Weighted mode	0.157	
	Heterogeneity tests	MR Egger	0.570	
		Inverse variance weighted	0.569	
	Horizontal Pleiotropy	MR Egger regression intercept	0.445	
		MR-PRESSO global test	0.670	
	Mendelian Randomization result	MR Egger	0.910	FGR
		Weighted median	0.212	
		Inverse variance weighted	0.048	
		Simple mode	0.497	
		Weighted mode	0.336	
	Heterogeneity tests	MR Egger	0.261	
		Inverse variance weighted	0.367	
	Horizontal Pleiotropy	MR Egger regression intercept	0.612	
		MR-PRESSO global test	0.496	
TGFB1	Mendelian Randomization result	MR Egger	0.465	FGR
		Weighted median	0.060	
		Inverse variance weighted	0.011	
		Simple mode	0.179	
		Weighted mode	0.248	
	Heterogeneity tests	MR Egger	0.228	
		Inverse variance weighted	0.357	
	Horizontal Pleiotropy	MR Egger regression intercept	0.708	
		MR-PRESSO global test	0.455	
TNFAIP3	Mendelian Randomization result	MR Egger	0.532	PE
		Weighted median	0.092	
		Inverse variance weighted	0.043	
		Simple mode	0.110	
		Weighted mode	0.343	
	Heterogeneity tests	MR Egger	0.780	
		Inverse variance weighted	0.791	
	Horizontal Pleiotropy	MR Egger regression intercept	0.536	
		MR-PRESSO global test	0.796	
	Mendelian Randomization result	MR Egger	0.241	FGR
		Weighted median	0.008	
		Inverse variance weighted	0.001	
		Simple mode	0.171	
		Weighted mode	0.115	
	Heterogeneity tests	MR Egger	0.841	
		Inverse variance weighted	0.854	
	Horizontal Pleiotropy	MR Egger regression intercept	0.576	
		MR-PRESSO global test	0.845	
TP53	Mendelian Randomization result	MR Egger	0.290	PTB
		Weighted median	0.043	
		Inverse variance weighted	0.027	
		Simple mode	0.204	
		Weighted mode	0.187	
	Heterogeneity tests	MR Egger	0.840	
		Inverse variance weighted	0.832	
	Horizontal Pleiotropy	MR Egger regression intercept	0.625	
		MR-PRESSO global test	0.554	
UBE2C	Mendelian Randomization result	MR Egger	0.580	FGR
		Weighted median	0.005	
		Inverse variance weighted	0.010	
		Simple mode	0.147	
		Weighted mode	0.133	
	Heterogeneity tests	MR Egger	0.103	
		Inverse variance weighted	0.262	
	Horizontal Pleiotropy	MR Egger regression intercept	0.937	
		MR-PRESSO global test	-	
UBE2G2	Mendelian Randomization result	MR Egger	0.586	GDM
		Weighted median	0.197	
		Inverse variance weighted	0.038	
		Simple mode	0.604	
		Weighted mode	0.666	
	Heterogeneity tests	MR Egger	0.598	
		Inverse variance weighted	0.747	
	Horizontal Pleiotropy	MR Egger regression intercept	0.821	
		MR-PRESSO global test	0.672	
UBE3 A	Mendelian Randomization result	MR Egger	0.587	PTB
		Weighted median	0.107	
		Inverse variance weighted	0.045	
		Simple mode	0.377	
		Weighted mode	0.370	
	Heterogeneity tests	MR Egger	0.840	
		Inverse variance weighted	0.832	
	Horizontal Pleiotropy	MR Egger regression intercept	0.670	
		MR-PRESSO global test	-	
USP21	Mendelian Randomization result	MR Egger	0.610	PTB
		Weighted median	0.116	
		Inverse variance weighted	0.049	
		Simple mode	0.643	
		Weighted mode	0.179	
	Heterogeneity tests	MR Egger	0.434	
		Inverse variance weighted	0.600	
	Horizontal Pleiotropy	MR Egger regression intercept	0.919	
		MR-PRESSO global test	0.648	

**Fig. 2:**
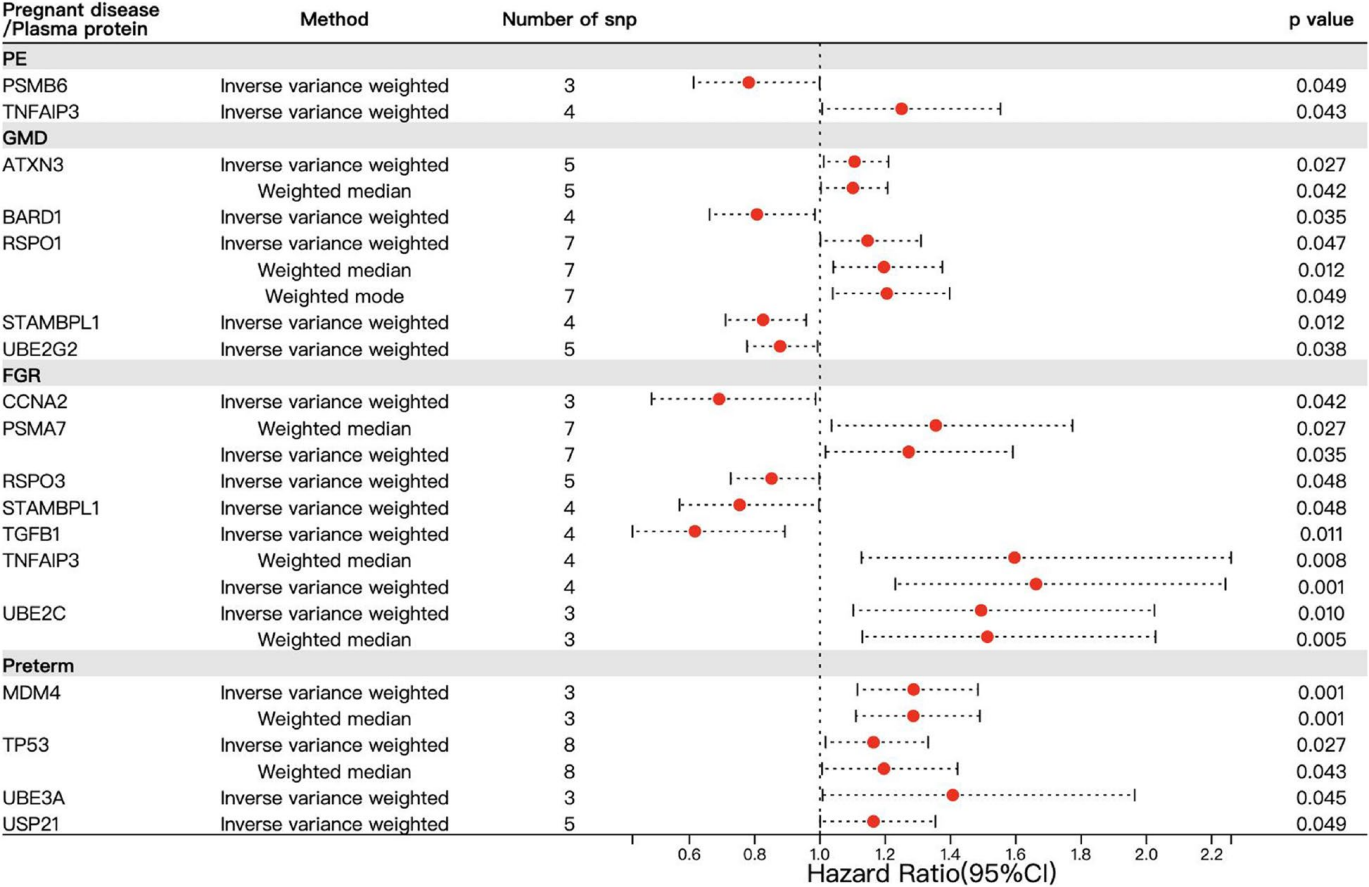
Results of MR analysis when ubiquitination related plasma proteins as outcome and pregnant diseases as exposure

Collectively, a total of four diseases with 18 proteins were found to be correlated with ubiquitination-associated protein regulation (summarized in Fig. 2), including:

- ①PE: PSMB6, and TNFAIP3;
- ②GDM: ATXN3, BARD1, RSPO1, STAMBPL1, and UBE2G2;
- ③FGR: CCNA2, PSMA7, RSPO3, STAMBPL1, TGFB1, TNFAIP3, and UBE2C;
- ④premature birth (PTB): MDM4, TP53, UBE3 A, and USP21.

### PE

**Fig. 3:**
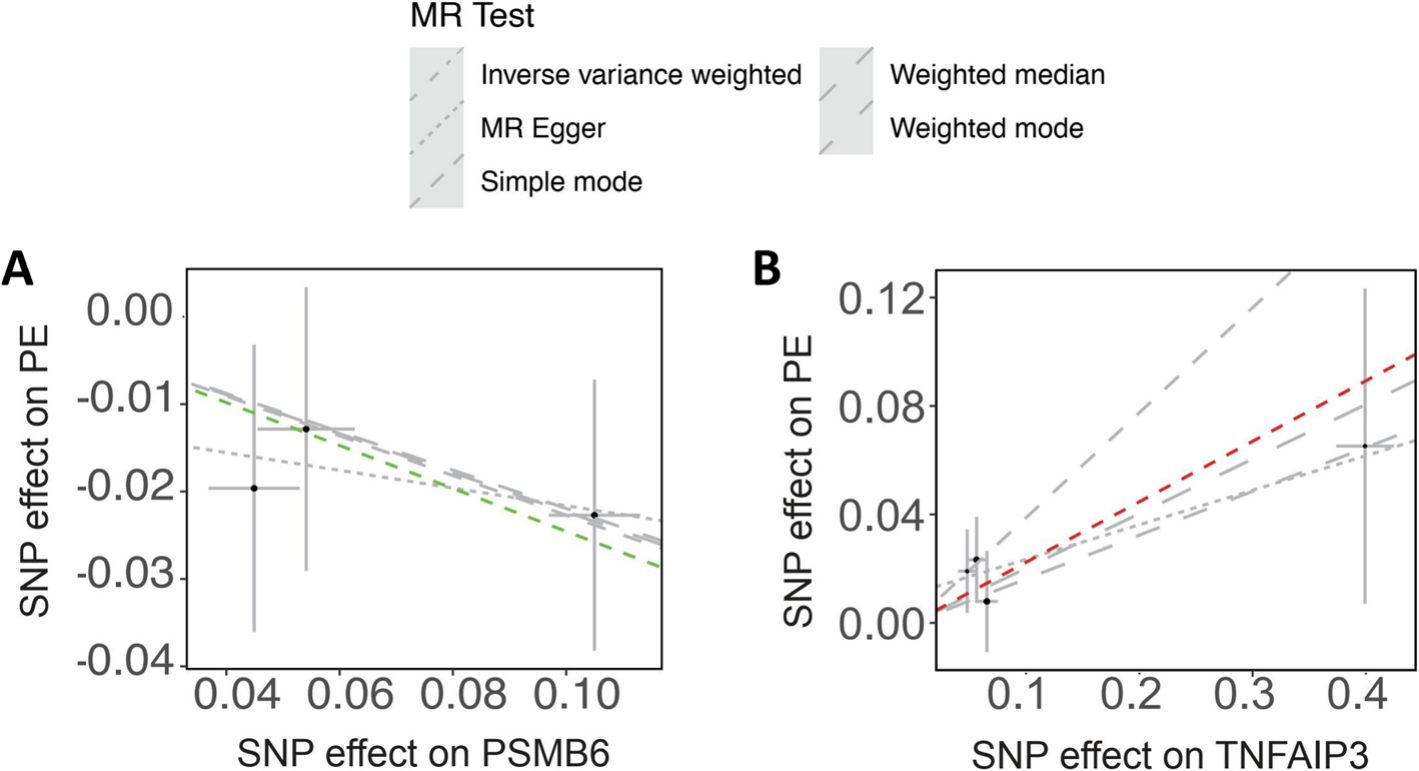
Identification of ubiquitination-associated protein regulation in PE by Mendelian randomization analysis

Proteasome subunit Y (PSMB6) is a component of the 20S core proteasome complex and participates in ubiquitin-dependent protein degradation within the 26S proteasome (Lichter et al. 2012). This complex maintains protein homeostasis by eliminating misfolded or unnecessary proteins. The expression of PSMB6 was found to be decreased in preeclamptic placentas (Bennani-Baiti et al. 2015), linking the ubiquitin-dependent protein degradation with PE. Our data of Mendelian randomization analysis showed that, PSMB6 is identified to be associated with PE, with 3 SNPs and *P* < 0.05 (*P* = 0.049) using IVW method (Figs. 2, 3A).

TNFAIP3 (TNF alpha induced protein 3) was identified as a tumor necrosis factor (TNF)-related gene whose expression is rapidly induced by TNF. A recent study reported that the transcription regulation region (TRR) of TNFAIP3 showed a hypomethylation with induction of 5-aza-CdR, which facilitated CREB recruitment and thereby participated in regulating trophoblast fusion (Yang et al. 2024), suggesting TNFAIP3 may be involved in syncytiotrophoblast stress, the first stage of the two-stage model of PE. Our data of Mendelian randomization analysis showed that, TNFAIP3 is identified to be associated with PE, with 4 SNPs and *P* < 0.05 (*P* = 0.043) using IVW method (Figs. 2, 3B).

### GDM

**Fig. 4:**
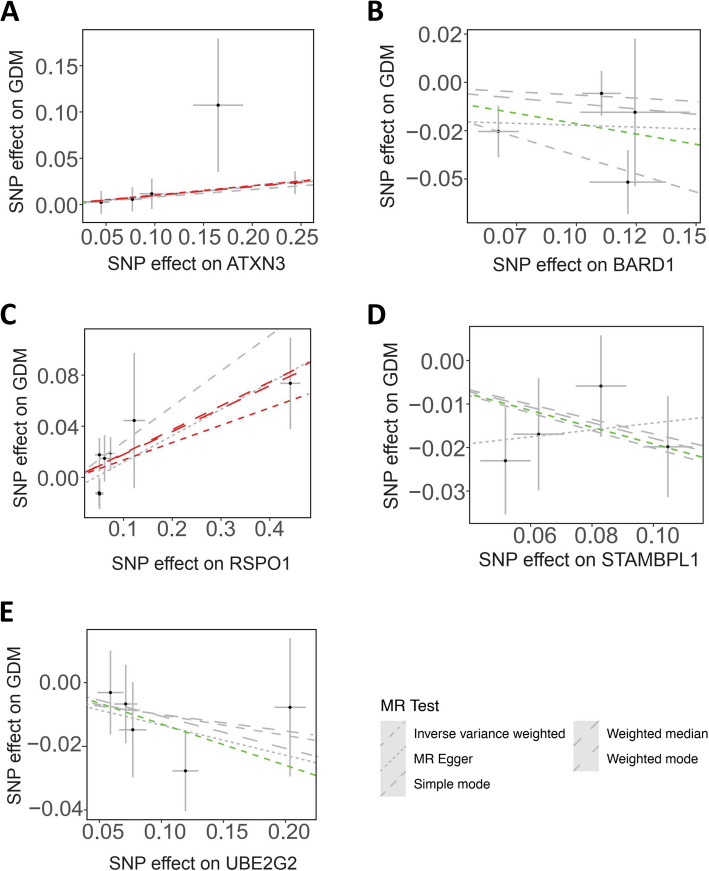
Identification of ubiquitination-associated protein regulation in GDM by Mendelian randomization analysis

The ubiquitin-specific peptidase Ataxin-3 (ATXN3), belonging to the Josephin family of deubiquitinases, is highly conserved and ubiquitously expressed in mammals (Wang et al. 2023). Physiological functions of ATXN3 presumably include ubiquitin protease and transcriptional corepressor activity, while inactivation of *Atxn3* gene increases protein ubiquitination in mice (Schmitt et al. 2007). A recent study showed that ATXN3 deubiquitinates and stabilizes the transcription coactivator Yes-associated protein (YAP) in a proteasome-dependent manner (Wu et al. 2023), while YAP influences β cell proliferation and diabetes (Nambiar et al. 2023), suggesting the potential role of ATXN3 in GDM. Our data of Mendelian randomization analysis showed that, ATXN3 is identified to be associated with GDM, with 5 SNPs and *P* < 0.05 (*P* = 0.027) using IVW method and 5 SNPs and *P* < 0.05 (*P* = 0.042) using weighted median method (Figs. 2, 4A).

The BReast CAncer type 1 susceptibility protein (BRCA1)-associated RING domain 1 (*BARD1*) gene encodes a 777-aa protein (Hawsawi et al. 2022; Witus et al. 2021), which usually functions by forming a complex with its heterodimeric binding partner BRCA1. The BRCA1/BARD1 complex localizes to damaged chromatin after DNA replication and catalyzes the ubiquitylation of histone H2 A and other cellular targets (Hu et al. 2021). The sole enzymatic function of the BRCA1/BARD1 complex is as a RING-type E3 ubiquitin (Ub) ligase, leading to the deposition of Ub signals onto a variety of substrate proteins. For example, ubiquitination of NF2 by the BRCA1/BARD1 complex in proliferating cells inhibits NF2/LATS association and Hippo signaling, resulting in the up-regulated stability of YAP protein that correlates positively with cell proliferation (Verma et al. 2019). Similarly, ubiquitin–proteasome degradation pathway plays a significant role in the coordinated protein stability of BRCA1 and its partner BARD1 in the female-specific ovarian granulosa cells. The proteasome-mediated degradation of BRCA1/BARD1 also occurs during the cAMP-dependent steroidogenic process, indicating the dynamic changes of BRCA1/BARD1 protein stability in ovarian granulosa cells provide an excellent paradigm for investigating the regulation of this protein complex under physiological conditions (Lu et al. 2007). A recent study reported that miR-210-3p was overexpressed in the pancreas of a GDM mouse model, where miR-210-3p negatively regulated BARD1 expression to exert protective effects on endometrial stromal cells against oxidative stress damage (Cao et al. 2022), suggesting the involvement of BARD1 in GDM. Our data of Mendelian randomization analysis showed that, BARD1 is identified to be associated with GDM, with 4 SNPs and *P* < 0.05 (*P* = 0.035) using IVW method (Figs. 2, 4B).

R-spondin1 (RSPO1), belonging to the R-spondin family, is expressed during early ovary development and has recently emerged as an important mediator of ovary development through activating the Wnt/β-catenin signaling, leading to suppression of testis formation (Tomaselli et al. 2011). Given the hormones secreted by female’s ovaries are pivotal for placentation and subsequently pregnant maintenance, it can be hypothesized that RSPO1 plays an important role during pregnancy. Wnt signaling downstream mutations have been implicated to be associated with fat distribution, and a recent study identifies that 12 obese patients harbor the same mutations in RSPO1 (p.R219 W/Q) predisposing to human obesity by screening all Wnt-related paracrine factors in 1994 obese cases and 2161 controls using whole-exome sequencing (WES) (Sun et al. 2023). Consistently, in another study by Kang et al*.*, it is reported that circulating RSPO1 levels are increased to a greater extent in the obese group than in the lean group, and serum levels of RSPO1 are higher in the insulin-resistant group than in the insulin-sensitive group (Kang et al. 2019), indicating that gain-of-function mutations and excessive expression of RSPO1 in a pregnant woman could be a risk factor contributing to obesity and GDM. Our data of Mendelian randomization analysis showed that, RSPO1 is identified to be associated with GDM, with 7 SNPs and *P* < 0.05 (*P* = 0.047) using IVW method, 7 SNPs and *P* < 0.05 (*P* = 0.012) using weighted median method, and 7 SNPs and *P* < 0.05 (*P* = 0.049) using weighted mode method (Figs. 2, 4C).

STAMBPL1, a deubiquitinating enzyme that cleaves Lys63 ubiquitin linkage (McCullough et al. 2004), is also associated with GDM. STAMBPL1 protein harbors nuclear localization signal (NLS), MPN (Mprl/Padl N-terminal) and JAMM (Jab1/MPN domain metalloenzyme) domains, thus belonging to the JAMM deubiquitinating enzyme family. It is found that STAMBPL1-E292 A (a mutant without deubiquitinating activity) significantly suppressed MKP1 protein polyubiquitination (Liu et al. 2022b), indicating the function of STAMBPL1 as a deubiquitinating enzyme (DUB) is dependent on its E292 aa residue. Our data of Mendelian randomization analysis showed that, STAMBPL1 is identified to be associated with GDM, with 4 SNPs and *P* < 0.05 (*P* = 0.012) using IVW method (Figs. 2, 4D).

E2 Ubiquitin Conjugase G2 (UBE2G2), acting as an E2 enzyme, functions as part of the endoplasmic reticulum (ER)-associated degradation (ERAD) pathway responsible for identification and degradation of misfolded proteins in the ER (Ju et al. 2010). UBE2G2 is found to be involved in proinsulin degradation and subsequent presentation of the PPIB10-18 autoantigen, which is involved in the generation of insulin-derived peptides, emphasizing the importance of proinsulin processing in the ER to Type 1 diabetes (T1D) pathogenesis and identify novel targets for future T1D therapies (Cremer et al. 2024). Given T1D is caused by destruction of pancreatic beta cells, it can be hypothesized that pregnant women with T1D would have similar clinical symptoms to GDM during gestation. Our data of Mendelian randomization analysis showed that, UBE2G2 is identified to be associated with GDM, with 5 SNPs and *P* < 0.05 (*P* = 0.038) using IVW method (Figs. 2, 4E).

### FGR

**Fig. 5:**
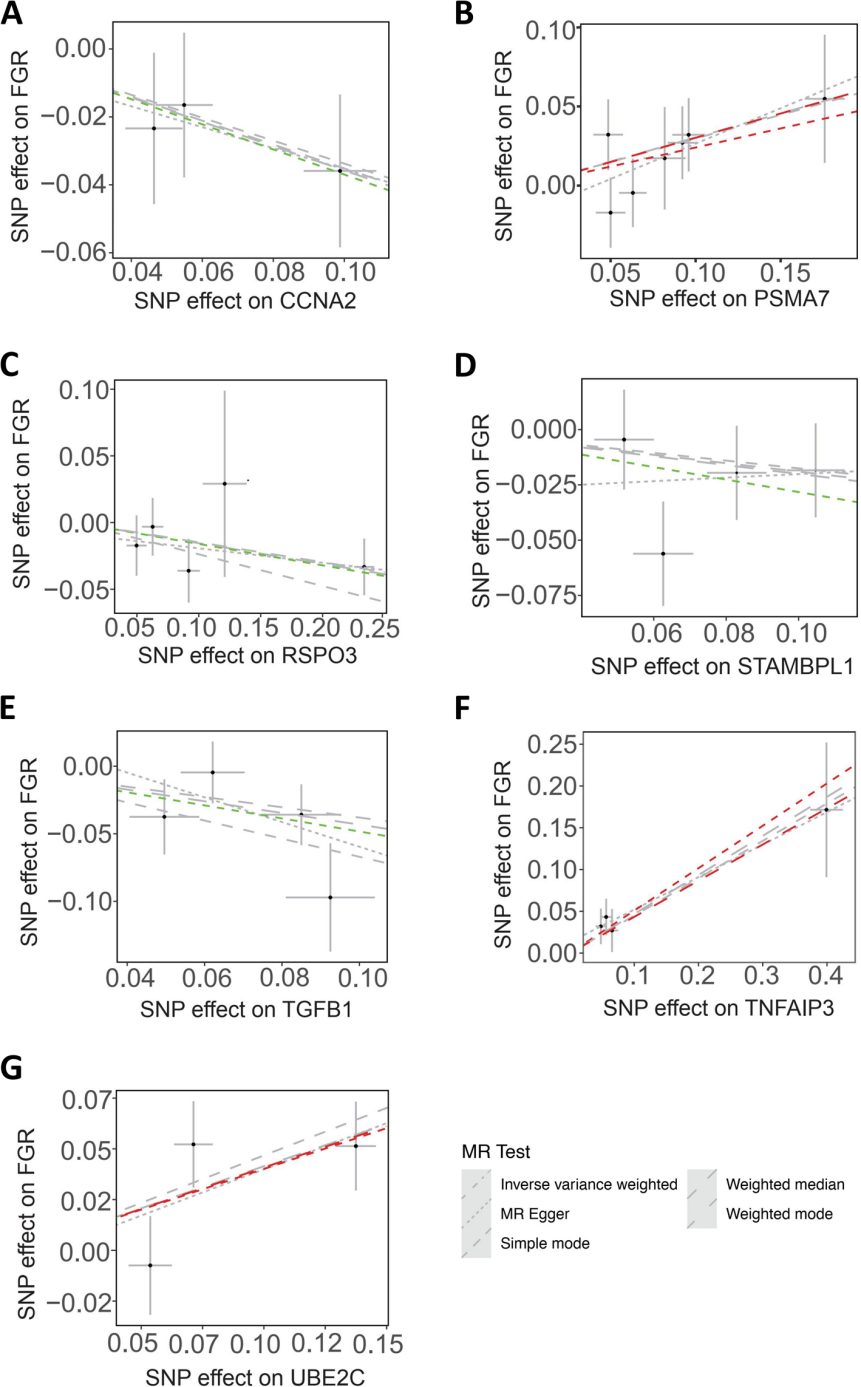
Identification of ubiquitination-associated protein regulation in FGR by Mendelian randomization analysis

Cyclin A2 (CCNA2, also known as CCNA, encoded by human *CCNA2* gene), belongs to the highly conserved cyclin family, whose members oscillate to regulate the cell cycle, and regulates the cell cycle by inducing transition through G1/S and G2/M through binding and interacting with cyclin-dependent kinases such as CDK1 and CDK2. CCNA2 expression was observed to be downregulated in trophoblast of Recurrent miscarriage (RM) first- trimester villi, and CCNA2 promotes migration of HTR8/SVneo cells via the RhoA-ROCK signaling, and increases HTR8/SVneo cells proliferation and inhibits their apoptosis via the p53 pathway (Li et al. 2019a), whereas silencing of CCNA2 repressed cell migration and invasion (Li et al. 2019b). A previous study reported that CCNA2 is essential for uterine function and fertility (Aljubran, et al. 2024), and in addition to its function in regulating the cell cycle, CCNA2 also directs E2 and P4 signaling in vitro. The CCNA2 expression was detected to be markedly decreased in endometrial tissues, particularly in the stromal cells from the women undergoing fertilization in vitro who failed to achieve a successful pregnancy (Aljubran, et al. 2024). Data from *Ccna2* uterine tissue-conditional knockout mouse revealed that, loss of *Ccna2* led to an inability to achieve pregnancy which appears to be due to alterations in the process of decidualization (Aljubran, et al. 2024). Our data of Mendelian randomization analysis showed that, CCNA2 is identified to be associated with FGR, with 3 SNPs and *P* < 0.05 (*P* = 0.042) using IVW method (Figs. 2, 5A).

The proteasome 20S subunit α7 (PSMA7), functioning as an α-type subunit of the 20S proteasome core particle that is involved in the regulated degradation of proteins in cells via the ubiquitin–proteasome system (Ren et al. 2019), has been proved to be a target interacting with some important proteins involved in transcription factor regulation (Du et al. 2009). A recent study further demonstrated that PSMA7 participates in degrading proteins through ubiquitin (Ub)-proteasome pathway (UPP) that plays an important role in the regulation of cell proliferation or cell cycle control, transcriptional regulation, immune and stress response, cell differentiation, and apoptosis (Du et al. 2009). Our data of Mendelian randomization analysis showed that, PSMA7 is identified to be associated with FGR, with 7 SNPs and *P* < 0.05 (*P* = 0.027) using weighted median method, and 7 SNPs and *P* < 0.05 (*P* = 0.035) using IVW method (Figs. 2, 5B).

R-spondin 3 (RSPO3), a member of the R-spondin family, plays a key role in development and tissue homeostasis (Niu, et al. 2018). A previous study reveals that the fracture reducing allele at the RSPO3 locus is associated with upregulated mRNA and protein expression levels of RSPO3, giving rise to the increased trabecular bone mineral density and the reduced risk of distal forearm fractures in humans (Nilsson et al. 2021), indicating the important role of RSPO3 in bone development. Moreover, pretreatment with RSPO3 could ameliorate the oxygen and glucose deprivation (OGD)/re-oxygenation (OGD/R)-induced neuronal cell death and oxidative injury by activating the Akt-Erk-β-Catenin signaling cascade, which is observed in both neuronal cells (Neuro-2a) and primary murine cortical neurons (Liu et al. 2023), suggesting the protective role of RSPO3 against hypoxia and also indicating the possible funcion of RSPO3 in fetal growth. In addition to a previous study showing *Cynoglossus semilaevis* Rspo3 mediates embryo development through deactivating the Wnt/β-Catenin signaling (Niu, et al. 2018), more recently, Shinozuka et al. showed that Rspo3 is dispensable for multiple developmental processes involving roof plate-derived Wnt ligands (Shinozuka et al. 2024), suggesting the pivotal regulation of Rspo3 through Wnt in fetal development. Our data of Mendelian randomization analysis showed that, RSPO3 is identified to be associated with FGR, with 5 SNPs and *P* < 0.05 (*P* = 0.048) using IVW method (Figs. 2, 5C).

STAM-binding-protein-like 1 (STAMBPL1, also known as AMSH-LP) shares 68% sequence identity and nearly identical folding of the catalytic JAB1/MPN/Mov34 metalloproteases (JAMMs) domains, as well as 56% overall sequence identity with STAMBP. As discussed above, STAMBPL1 expression is also associated with GDM in addition to FGR, it can be thereby naturally hypothesized that STAMBPL1 exerts protective effects on pregnant women during gestation. Acting as a deubiquitinase, STAMBPL1 plays a pivotal role in endocytic trafficking and lysosomal degradation (Wang, et al. 2022), and is also a positive regulator of Tax activation of NFB (Lee et al. 2017). Our data of Mendelian randomization analysis showed that, STAMBPL1 is identified to be associated with FGR, with 4 SNPs and *P* < 0.05 (*P* = 0.048) using IVW method (Figs. 2, 5D).

Transforming growth factor beta1 (TGFB1) is involved in selective intrauterine growth restriction (sIUGR, also known as FGR), which specifically occurs in monochorionic (MC) twins, usually has a poor prognosis and the underlying mechanisms are not well understood. The ten-eleven translocation 2 (TET2)-mediated DNA hydroxymethylation of TGFB1 is demonstrated to be associated with sIUGR in monochorionic twin pregnancies (Jiang et al. 2023). Our data of Mendelian randomization analysis showed that, TGFB1 is identified to be associated with FGR, with 4 SNPs and *P* < 0.05 (*P* = 0.011) using IVW method (Figs. 2, 5E).

The Tumor Necrosis Factor Alpha-Induced Protein 3 (TNFAIP3, also known as the intracellular ubiquitin-editing protein A20) is a cytoplasmic protein that plays a key role in the negative regulation of inflammation and immunity (Verstrepen et al. 2010). The N-terminal half of TNFAIP3 protein encodes a deubiquitinating (DUB) domain, whereas the C-terminal half encodes a zinc finger-containing E3 ligase domain. These two enzymatic activities work together to control the ubiquitination and subsequent degradation of cellular substrates (Wertz et al. 2004). Previous studies showed that TNFAIP3 expression is obviously induced by multiple stimuli, including the proinflammatory cytokines TNFα and IL-1, and microbial products that trigger pathogen recognition receptors, such as Toll-like receptors, while TNFAIP3 suppresses TNFα-induced signaling pathways (Turner 2006). In addition, TNFAIP3 is found to potentiate the deubiquitination of K63-polyubiquitin chains either directly through its N-terminal deubiquitinase domain or by disrupting the interaction between E3 and E2 enzymes that catalyze K63-polyubiquitination (Kolodziej et al. 2011). Our data of Mendelian randomization analysis showed that, TNFAIP3 is identified to be associated with FGR, with 4 SNPs and *P* < 0.05 (*P* = 0.008) using weighted median method, and 7 SNPs and *P* < 0.05 (*P* = 0.001) using IVW method (Figs. 2, 5F).

Ubiquitin-conjugating enzyme E2 C (UBE2C) regulates ubiquitylation chain formation via the K11 linkage in ubiquitin-proteosome system (Zhang, et al. 2023). UBE2C is found to be highly expressed in a variety of cancer types, which is strongly associated with an unfavorable prognosis, and is implicated in the progression of various cancers (Li, et al. 2024; Chiang, et al. 2020; Wang et al. 2017). For example, in lung cancer, UBE2C expression is induced by KRAS-G12D and UBE2C promotes ubiquitylation and degradation of DEPTOR, causing mTORC activation (Zhang, et al. 2023). In addition, UBE2C-triggered sodium-coupled neutral amino acid transporter 2 (SNAT2) monoubiquitination at lysine 59 inhibits K63-linked polyubiquitination at lysine 33 of SNAT2, resulting in the consequently lymphangiogenesis and lymph node (LN) metastasis in patients with bladder cancer (BCa). Despite its function in carcinogenesis, the potential role of UBE2C in placentation and pregnant maintenance needs further investigation. Our data of Mendelian randomization analysis showed that, UBE2C is identified to be associated with FGR, with 3 SNPs and *P* < 0.05 (*P* = 0.010) using IVW method, and 4 SNPs and *P* < 0.05 (*P* = 0.005) using weighted median method (Figs. 2, 5G).

### Preterm birth (PTB)

**Fig. 6:**
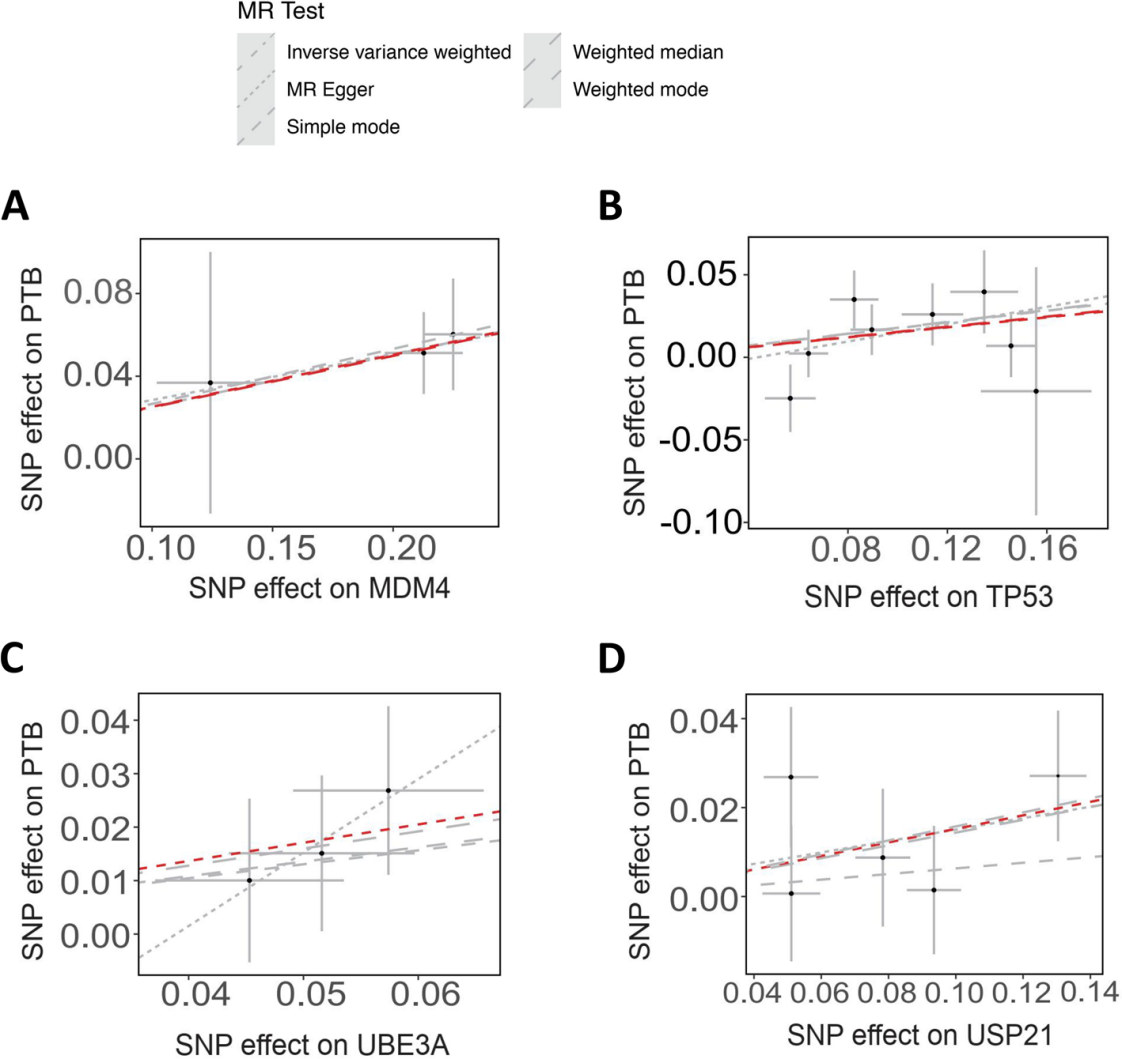
Identification of ubiquitination-associated protein regulation in PTB by Mendelian randomization analysis

Originally cloned in 1996 from mouse and in 1997 from human, the Mouse double minute 4 (MDM4), also known as MDMX or HDMX (human MDMX), is a critical negative regulator of the tumor suppressor p53. As a member in MDM protein family, MDM4 was identified to act as a critical regulator of p53 in cancer (Markey 2011). Recently, studies have revealed that MDM4 is also associated with PB occurrence and development. Mei et al*.* reported that pharmacological inhibition of MDM4 with XI-011, a small molecular inhibitor of MDM4 that significantly reduced MDM4 expression and increased the expression of total and acetylated p53, could effectively alleviate pulmonary fibrosis through the MDM4-p53-dependent pathway (Mei et al. 2023), suggesting the potential role of MDM4 in fetal lung development and growth. Another study showed that, the expression of let-7 microRNA (miRNA) was obviously up-regulated in PTB-associated placental tissues, and let-7 regulated EVT cells proliferation, migration and invasion via targeting MDM4 (Zhang et al. 2019), reinforcing the notion that MDM4 would be a promising target for PTB prediction and diagnosis. Our data of Mendelian randomization analysis showed that, MDM4 is identified to be associated with PTB, with 3 SNPs and *P* < 0.05 (*P* = 0.001) using IVW method, and 3 SNPs and *P* < 0.05 (*P* = 0.001) using weighted median method (Figs. 2, 6A).

Transformation-related protein 53 (Trp53), which encodes the p53 protein, functions as a tumor suppressor gene whose mutation is strongly associated with cancer (Hirota et al. 2010). A previous study revealed that deficiency of uterine-specific *p53* confers premature uterine senescence and promotes PTB in mice. Recently, Jiang et al*.* reported the long non-coding RNA SNHG29 was overexpressed in the placentas of women who delivered preterm with labor, and the elevated expression of SNHG29 regulates HTR8/SVneo cell senescence via the p53/p21 signaling in spontaneous PTB (Jiang et al. 2021), indicating the increased pro-inflammatory cytokine expression and the subsequent release by senescent cells would be pivotal to the pathophysiology of spontaneous PTB. Similarly, either the antidiabetic drug metformin or the antioxidant resveratrol that activating the AMPK signaling protected against spontaneous and inflammation-induced PTB in *p53*-deleted female mice (Deng et al. 2016), further suggesting that metformin and resveratrol have therapeutic potential to prevent PTB. Our data of Mendelian randomization analysis showed that, TP53 is identified to be associated with PTB, with 8 SNPs and *P* < 0.05 (*P* = 0.027) using IVW method, and 8 SNPs and *P* < 0.05 (*P* = 0.043) using weighted median method (Figs. 2, 6B).

Ubiquitin-protein ligase E3 A (UBE3 A) acts as an E3 ubiquitin ligase whose dysregulation has been implicated in autism and Angelman syndrome (AS, an incurable neurodevelopmental disease) (Dindot, et al. 2023; Maranga et al. 2020). Mechanistically, it is reported that UBE3 A promotes synapse elimination through suppressing activity of the BMP signaling (Furusawa et al. 2023), proving the essential role of UBE3 A during fetal neurodevelopment. Our data of Mendelian randomization analysis showed that, UBE3 A is identified to be associated with PTB, with 3 SNPs and *P* < 0.05 (*P* = 0.045) using IVW method (Figs. 2, 6C).

As a proteasome-associated deubiquitinating enzyme that is responsible for intracellular protein degradation, the Ubiquitin-specific protease 21(USP21) plays an important role in during oocyte maturation (Rong et al. 2022), and a recent study additionally reported that the expression of USP21 was reduced in centrosomal protein of 78 kDa (*Cep78*)-knockout mice, leading to male infertility (Zhang, et al. 2022), while its function in pregnant maintenance remains to be investigated. Our data of Mendelian randomization analysis showed that, USP21 is identified to be associated with PTB, with 5 SNPs and *P* < 0.05 (*P* = 0.049) using IVW method (Figs. 2, 6D).

## Targeting ubiquitination as potential therapeutic strategy for diseases during pregnancy

Ubiquitination targeting therapy for perinatal diseases refers to the use of ubiquitination targeting to regulate and treat perinatal related diseases. Currently, the research on ubiquitination targeting therapy for perinatal diseases mainly focuses on the following two aspects (Cruz Walma et al. 2022): 1) Target identification; and 2) Drug development.

### Target identification

By studying the pathogenesis and related signaling pathways of perinatal diseases, protein targets closely related to disease occurrence and development can be identified. These targets may be factors that promote disease progression or inhibit disease progression, which have been discussed above and summarized in Table 3.

### Drug development

Based on identified targets, drug molecules can be designed and synthesized to intervene in the occurrence and development of certain diseases by regulating the modification levels of ubiquitination targets. These developed drugs can be molecules that promote ubiquitination or inhibit ubiquitination, depending on the characteristics of the target. For example, recent studies have shown that E3 ubiquitin ligase, playing a key role in the process of fetal development and placental formation, affects the biological behavior of placental trophoblast cells and thereby causes a series of pregnancy complications that threaten the health of both mother and fetus (Feng et al. 2023). Silencing of Cullin 1, a member of Cullin protein family E3 ubiquitin ligase, significantly inhibits outgrowth of extravillous explants in vitro, as well as invasion and migration of HTR8/SVneo cells (Zeng et al. 2021), suggesting its role in PE occurrence. Thus, developing drugs targeting such molecules as Cullin 1 could be promising in intervention of related diseases. Up to now, the research on ubiquitination targeting therapy for perinatal diseases is still at the preliminary stage (Dai et al. 2024), but it has potential clinical application prospects. Through this treatment strategy, it is expected to provide new means for the prevention and treatment of perinatal diseases.

## Conclusion and perspectives

Ubiquitination refers to the process of adding small particles of ubiquitin to target proteins, and ubiquitin is known as a small protein that can bind to other proteins and label them for cellular processes such as degradation, repair, or transport. Due to the work in the past decades, ubiquitination has been identified to function as an important cellular regulatory mechanism involved in regulating many biological processes in human placenta, such as placental development, placental function, and placental activity (Cockram et al. 2021; Qin et al. 2022).

The development of human placenta is initiated by the implantation of the blastocyst, and the placenta is developed from the trophectoderm (TE). Placenta plays an essential role in maintaining and supporting the growth and development of the embryo during pregnancy, when placenta provides nutrients, oxygen, and immune protective substances to the fetus, and plays a filtering and excretory role, expelling waste from the fetus from the mother (Aplin et al. 2020). In addition, the placenta also secretes hormones that regulate the physiological status of the mother and fetus (Tang et al. 2023), resulting in a successful pregnancy. As we summarize herein, recent studies have shown that, as an important post-translational modification, the ubiquitination regulation plays an important role in placentation.

Ubiquitination participates in embryo implantation, placental formation and placental function during human placentation (Feng et al. 2023; Yin 2024). During the embryo implantation process, ubiquitination modification can regulate the timing and location of embryo implantation (Jiang et al. 2017). Accordingly, the functional deficiency/inactivation or overexpression/hyperactivation of certain ubiquitinase that modulate ubiquitination modification can lead to embryo implantation disorders. During placental formation, ubiquitination modification can regulate the interaction between embryonic and endometrial cells, thereby ensuring normal embryonic development and promoting placental development. Some studies regarding to ubiquitination enzymes and substrates have shown that ubiquitination modification plays a pivotal role in directing placental formation (Chen et al. 2015; Liu et al. 2021). As the border tissue between the embryo and the mother, the placenta plays multiple functions such as nutritional supply, metabolic regulation, and immune protection (Jin, et al. 2022). Ubiquitination modification can regulate the expression and degradation of multiple key proteins in placental cells (summarized in Table 1), thereby affecting the normal functioning of the placenta.

Currently, series of researches have elucidated that dysregulation of ubiquitination is closely associated with the occurrence and development of obstetric diseases or gestational disorders in clinic. For example, in certain pregnancy related diseases such as PE, GDM, FGR, and PTB that discussed in this review article, aberrant activity of the ubiquitination pathway may lead to abnormal degradation of some important proteins, leading to the deviant expression of those proteins including PSMB6, TNFAIP3, ATXN3, BARD1, RSPO1, STAMBPL1, UBE2G2, CCNA2, PSMA7, RSPO3, TGFB1, UBE2C, MDM4, TP53, UBE3 A, USP21 and consequently possible occurrence of the diseases (Chen et al. 2021b; Li et al. 2022; Aye et al. 2022). In addition, ubiquitination can also regulate some important signaling pathways related to placental development and function, such as cell growth- and cell cycle-associated signalings including the mTOR, AMPK, Wnt, TGF-beta, Hippo, RhoA-ROCK, TNF-alpha, BMP, and NF-B signalings, thereby affecting embryonic development and pregnancy outcomes.

Overall, in both cell biology and developmental biology research, ubiquitination and placenta play important roles in understanding cell regulation and embryonic growth and development processes. Particularly, ubiquitination regulation of placental development is a complex and critical process involving the interaction of multiple ubiquitination enzymes and substrates. Future research can further explore the deeply molecular mechanisms by which ubiquitination modification regulates placental development and identify potentially targeted treatment or intervention strategies.

## Supplementary Information

Supplementary Material 1. Table S1. Employed SNPs instruments in Mendelian randomization analysis.

## Data Availability

No datasets were generated or analysed during the current study.
